# The genome sequence of the Ochreous Pearl, *Anania crocealis *(Hübner, 1796)

**DOI:** 10.12688/wellcomeopenres.20573.1

**Published:** 2023-12-06

**Authors:** David C. Lees, Natural History Museum Genome Acquisition Lab, Darwin Tree of Life Barcoding collective, Wellcome Sanger Institute Tree of Life Management, Samples and Laboratory team, Wellcome Sanger Institute Scientific Operations: Sequencing Operations, Wellcome Sanger Institute Tree of Life Core Informatics team, Tree of Life Core Informatics collective, Darwin Tree of Life Consortium

**Affiliations:** 1 Natural History Museum, London, England, UK

**Keywords:** Anania crocealis, Ochreous Pearl, genome sequence, chromosomal, Lepidoptera

## Abstract

We present a genome assembly from an individual male *Anania crocealis* (the Ochreous Pearl; Arthropoda; Insecta; Lepidoptera; Crambidae). The genome sequence is 624.0 megabases in span. Most of the assembly is scaffolded into 29 chromosomal pseudomolecules, including the Z sex chromosome. The mitochondrial genome has also been assembled and is 15.33 kilobases in length. Gene annotation of this assembly on Ensembl identified 20,293 protein coding genes.

## Species taxonomy

Eukaryota; Metazoa; Eumetazoa; Bilateria; Protostomia; Ecdysozoa; Panarthropoda; Arthropoda; Mandibulata; Pancrustacea; Hexapoda; Insecta; Dicondylia; Pterygota; Neoptera; Endopterygota; Amphiesmenoptera; Lepidoptera; Glossata; Neolepidoptera; Heteroneura; Ditrysia; Obtectomera; Pyraloidea; Crambidae; Pyraustinae; *Anania*; *Anania crocealis* (Hübner, 1796) (NCBI:txid1100908).

## Background

The Ochreous Pearl, *Anania crocealis* (Hübner, 1796), is a crambid moth with a wingspan of about 22–25 mm ( Goater, 1986), forewing length 11–12 mm ( Sterling & Parsons, 2018), its forewings pale yellow with the very small reddish brown orbicular and reniform stigmata bounded by a convex basal transverse line and a distal transverse line which is concave around the outer stigma, and whitish grey hindwings; the terminal line in both wings is also reddish-brown. It is similar to a few other UK Pyraustinae such as *Paratalanta hyalinalis* (Hübner, 1796) as well and the whiter *Paracorsia repandalis* ([Denis & Schiffermüller], 1775] but differs in the relatively unmarked hindwings. Like many pyraustines, it has a triangular resting posture.

The adult moth flies in the UK from late June to August ( Goater, 1986; Parsons & Clancy, 2023), and the moth is regarded as univoltine or sometimes bivoltine flying up to September or even November in the south of the UK ( Parsons & Clancy, 2023; Sterling & Parsons, 2018). The adult moth is mainly nocturnal but easily disturbed around the foodplant by day.

The Ochreous Pearl has a preference for marshy and damp habitats in the UK as well as drier slopes on chalk where its larval foodplants occur (Common Fleabane, *Pulicaria dysenterica* (L.) Bernh., Ploughman’s Spikenard *Pentanema squarrosum* (L.) D.Gut.Larr., Santos-Vicente, Anderb., E.Rico & M.M.Mart.Ort. as well as, in Austria, *Buphthalmum salicifolium* L.; all Asteraceae; Kettner, 2023). The black headed larvae also with a black prothoracic plate and pale grey green with a darker green dorsal line, start out under a downturned leaf margin, later feeding among silk inside the shoots ( Parsons & Clancy, 2023; Sterling & Parsons, 2018).

*A. crocealis* is more or less common and widespread in southern parts of the United Kingdom, occurring more locally in Ireland, Wales, northwest England and Scotland where scarce, as well as Isle of Man and Channel Islands ( Parsons & Clancy, 2023). In Europe it occurs widely from western France to Eastern Europe, the far south of Scandinavia, as far as the eastern edge of the Black Sea, with a few records in north-west Africa (Morocco), north-eastern Spain, Italy, Greece, Croatia and western Russia ( GBIF Secretariat, 2023, BOLD 2/11/2023).

*A. crocealis* exhibits a single mitochondrial cluster on BOLD, BOLD:AAD7537 (2023-10-30). The mitogenome of the sequenced specimen (OX438878) is identical (658 bp) to the prevalent continental haplotype and a few other DNA barcodes from UK specimens are a single nucleotide different. *Anania testacealis* (Zeller, 1847) from the Mediterranean regions is around 1.2% divergent but belongs to the same BIN cluster; there is an intermediate sequence identified as *A. crocealis* DNA barcoded from Austria. Its closest relative otherwise is unclear; the nearest cluster on BOLD is BOLD:AAQ3746 (one specimen identified as *Pyrausta trimaculalis*) which is morphologically dissimilar and at least 5.26% divergent, but *Anania perlucidalis* (Hübner, [1809]), a species treated in Yang *et al*. (2012) is from 5.53% divergent. The type species of *Anania* Hübner, 1823 is *Pyralis guttalis* [Denis & Schiffermüller], 1775 ( *Anania funebris*). Although *Pyralis crocealis* Hübner, 1796 is the type species of *Ebulea* Doubleday 1849, several such genera were synonymised with *Anania* by Leraut (2005); see also Trankner *et al*. (2009). *Anania* exhibit an elongated, asymmetric sclerite of the phallus apodeme and female genitalia with a digitiform sclerotization inside the antrum ( Trankner *et al.*, 2009). The genus *Anania* Hübner, 1823 is currently placed in the tribe Pyraustini ( Mally *et al.*, 2019).

The genome data may be useful to investigate questions such as the closest relatives of *A. crocealis*, and adaptations to feed on Asteraceae: Inuleae.

The genome of the Ochreous Pearl, *Anania crocealis*, was sequenced as part of the Darwin Tree of Life Project, a collaborative effort to sequence all named eukaryotic species in the Atlantic Archipelago of Britain and Ireland. Here we present a chromosomally complete genome sequence for *Anania crocealis*, based on one male specimen from Sandwich Bay Bird Observatory, England.

## Genome sequence report

**Figure 1.**
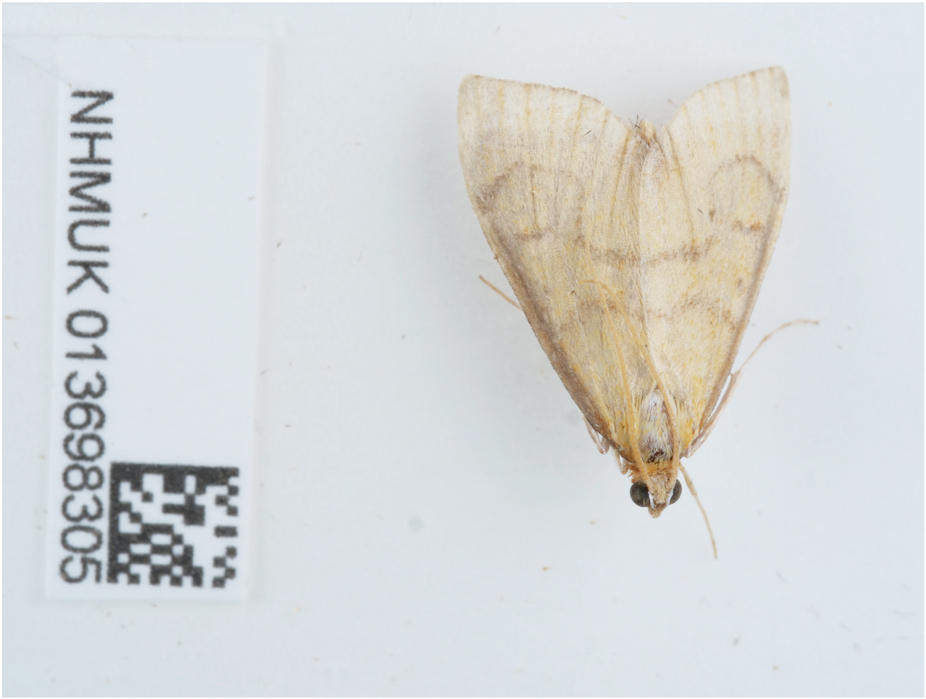
Photograph of the *Anania crocealis* (ilAnaCroc1) specimen used for genome sequencing.

The genome was sequenced from one male *Anania crocealis* ( Figure 1) (see methods). A total of 47-fold coverage in Pacific Biosciences single-molecule HiFi long reads was generated. Primary assembly contigs were scaffolded with chromosome conformation Hi-C data. Manual assembly curation corrected 12 missing joins or mis-joins and removed one haplotypic duplications, reducing the scaffold number by 7.69%.

**Figure 2.**
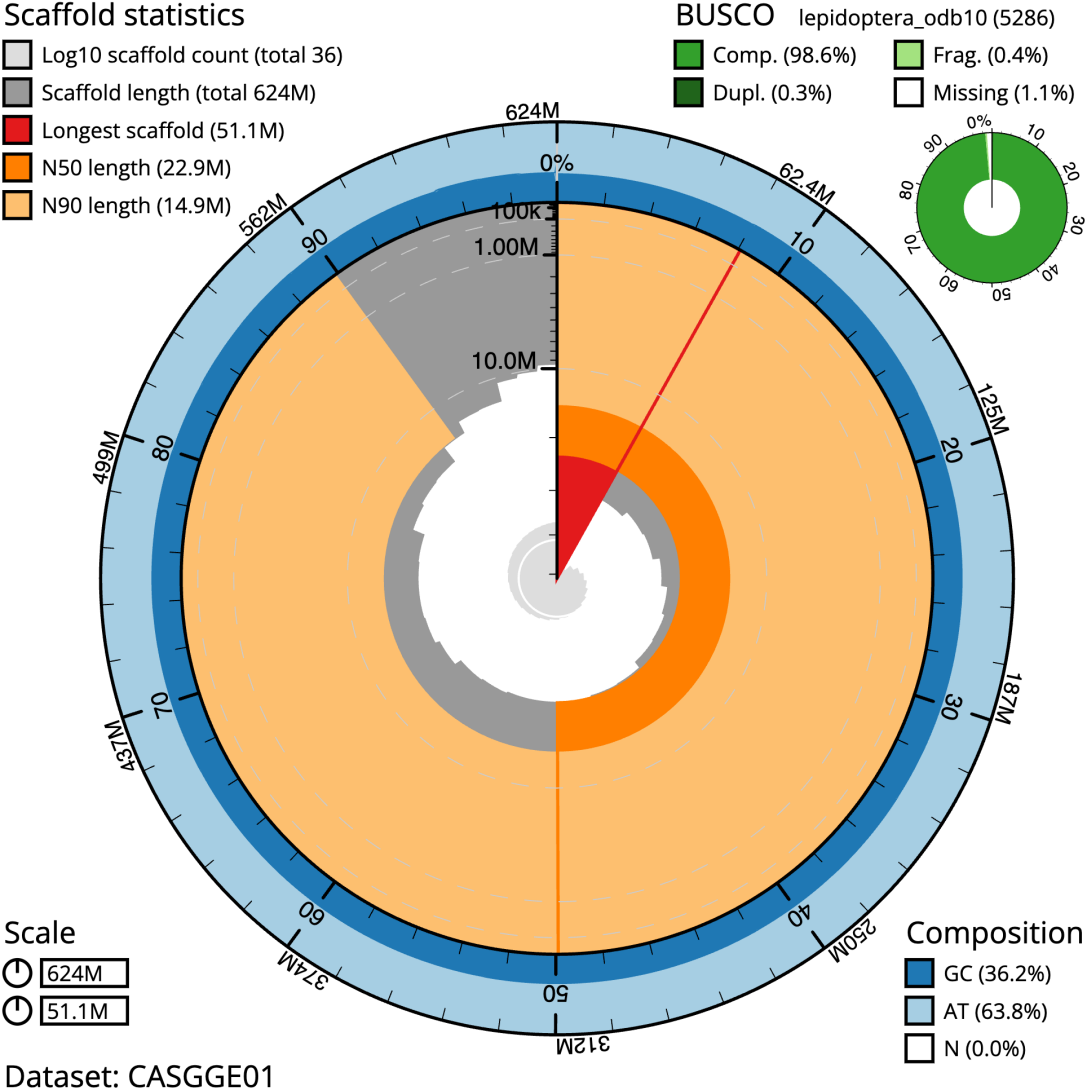
Genome assembly of *Anania crocealis*, ilAnaCroc1.1: metrics. The BlobToolKit Snailplot shows N50 metrics and BUSCO gene completeness. The main plot is divided into 1,000 size-ordered bins around the circumference with each bin representing 0.1% of the 623,994,487 bp assembly. The distribution of scaffold lengths is shown in dark grey with the plot radius scaled to the longest scaffold present in the assembly (51,121,332 bp, shown in red). Orange and pale-orange arcs show the N50 and N90 scaffold lengths (22,872,268 and 14,854,060 bp), respectively. The pale grey spiral shows the cumulative scaffold count on a log scale with white scale lines showing successive orders of magnitude. The blue and pale-blue area around the outside of the plot shows the distribution of GC, AT and N percentages in the same bins as the inner plot. A summary of complete, fragmented, duplicated and missing BUSCO genes in the lepidoptera_odb10 set is shown in the top right. An interactive version of this figure is available at https://blobtoolkit.genomehubs.org/view/Anania%20crocealis/dataset/CASGGE01/snail.

**Figure 3.**
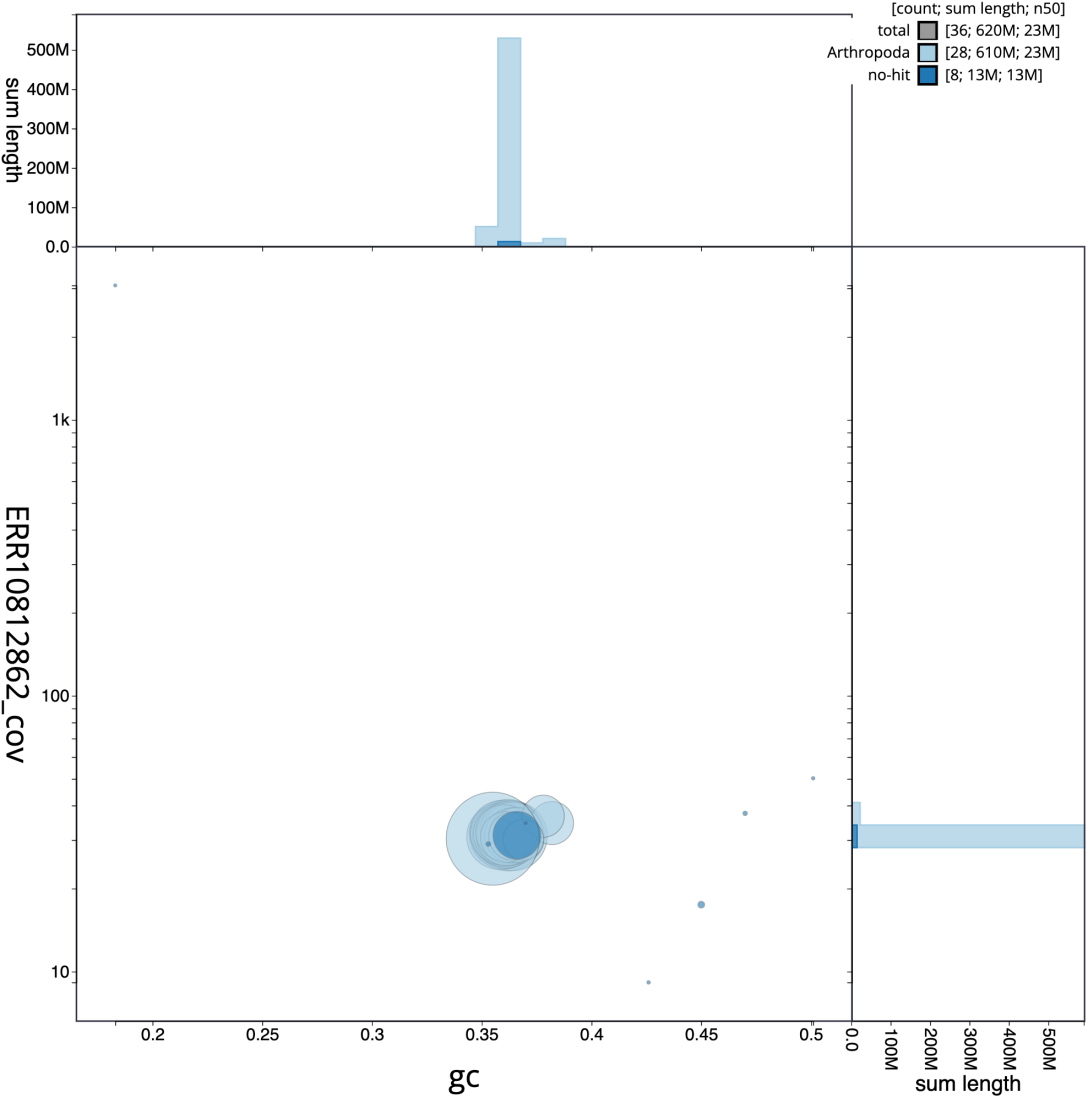
Genome assembly of *Anania crocealis*, ilAnaCroc1.1: BlobToolKit GC-coverage plot. Scaffolds are coloured by phylum. Circles are sized in proportion to scaffold length. Histograms show the distribution of scaffold length sum along each axis. An interactive version of this figure is available at https://blobtoolkit.genomehubs.org/view/Anania%20crocealis/dataset/CASGGE01/blob.

**Figure 4.**
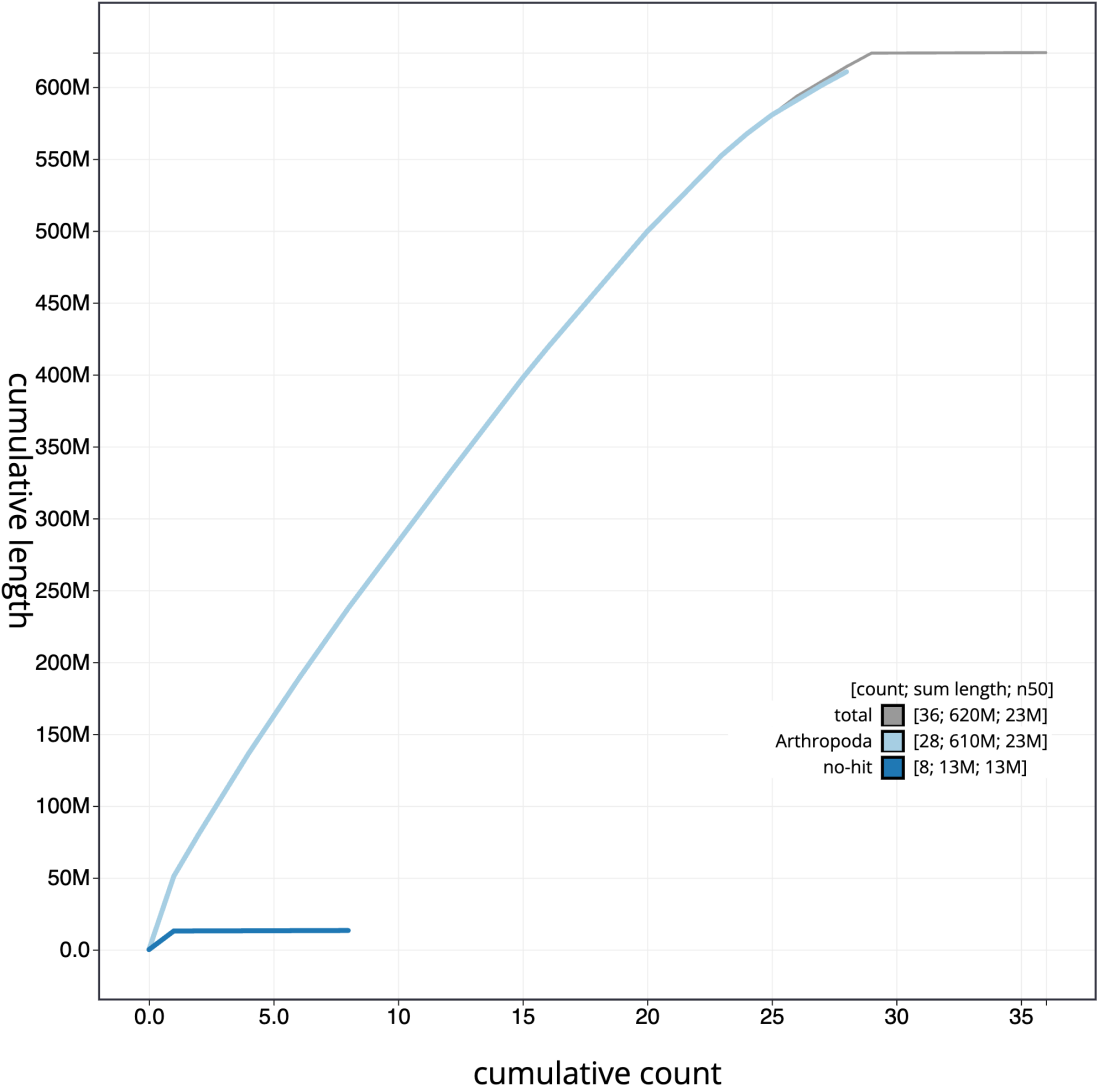
Genome assembly of *Anania crocealis*, ilAnaCroc1.1: BlobToolKit cumulative sequence plot. The grey line shows cumulative length for all scaffolds. Coloured lines show cumulative lengths of scaffolds assigned to each phylum using the buscogenes taxrule. An interactive version of this figure is available at https://blobtoolkit.genomehubs.org/view/Anania%20crocealis/dataset/CASGGE01/cumulative.

**Figure 5.**
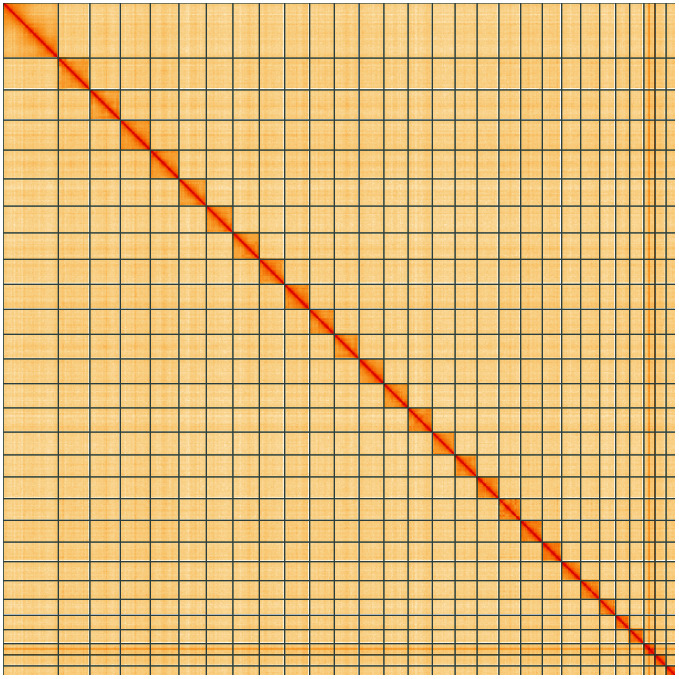
Genome assembly of *Anania crocealis*, ilAnaCroc1.1: Hi-C contact map of the ilAnaCroc1.1 assembly, visualised using HiGlass. Chromosomes are shown in order of size from left to right and top to bottom. An interactive version of this figure may be viewed at https://genome-note-higlass.tol.sanger.ac.uk/l/?d=AD5FhWJjTf2tlyGpTOyX_w.

The final assembly has a total length of 624.0 Mb in 35 sequence scaffolds with a scaffold N50 of 22.9 Mb ( Table 1). The snailplot in Figure 2 provides a summary of the assembly statistics, while the distribution of assembly scaffolds on GC proportion and coverage is shown in Figure 3. The cumulative assembly plot in Figure 4 shows curves for subsets of scaffolds assigned to different phyla. Most (99.95%) of the assembly sequence was assigned to 29 chromosomal-level scaffolds, representing 28 autosomes and the Z sex chromosome. Chromosome Z was assigned by alignment to *Eudonia lacustrata* (GCA_947562085.1) ( Boyes *et al.*, 2023). Chromosome-scale scaffolds confirmed by the Hi-C data are named in order of size ( Figure 5; Table 2). While not fully phased, the assembly deposited is of one haplotype. Contigs corresponding to the second haplotype have also been deposited. The mitochondrial genome was also assembled and can be found as a contig within the multifasta file of the genome submission.

**Table 1.** Genome data for *Anania crocealis*, ilAnaCroc1.1.

Project accession data		
Assembly identifier	ilAnaCroc1.1	
Species	*Anania crocealis*	
Specimen	ilAnaCroc1	
NCBI taxonomy ID	1100908	
BioProject	PRJEB59304	
BioSample ID	SAMEA111458574	
Isolate information	ilAnaCroc1, male: head and thorax (DNA sequencing and Hi-C scaffolding), abdomen (RNA sequencing)	
Assembly metrics *		*Benchmark*
Consensus quality (QV)	67.7	*≥ 50*
*k*-mer completeness	100%	*≥ 95%*
BUSCO **	C:98.6%[S:98.3%,D:0.3%],F:0.4%,M:1.1%,n:5,286	*C ≥ 95%*
Percentage of assembly mapped to chromosomes	99.95%	*≥ 95%*
Sex chromosomes	Z chromosome	*localised homologous pairs*
Organelles	Mitochondrial genome assembled	*complete single alleles*
**Raw data accessions**		
PacificBiosciences SEQUEL II	ERR10812862	
Hi-C Illumina	ERR10818324	
PolyA RNA-Seq Illumina	ERR12035179	
**Genome assembly**		
Assembly accession	GCA_949315895.1	
*Accession of alternate haplotype*	GCA_949316005.1	
Span (Mb)	624.0	
Number of contigs	99	
Contig N50 length (Mb)	14.9	
Number of scaffolds	35	
Scaffold N50 length (Mb)	22.9	
Longest scaffold (Mb)	51.1	
**Genome annotation**		
Number of protein-coding genes	20,293	
Number of gene transcripts	20,486	

* Assembly metric benchmarks are adapted from column VGP-2020 of “Table 1: Proposed standards and metrics for defining genome assembly quality” from ( Rhie *et al.*, 2021). ** BUSCO scores based on the lepidoptera_odb10 BUSCO set using v5.3.2. C = complete [S = single copy, D = duplicated], F = fragmented, M = missing, n = number of orthologues in comparison. A full set of BUSCO scores is available at https://blobtoolkit.genomehubs.org/view/Anania%20crocealis/dataset/CASGGE01/busco.

**Table 2.** Chromosomal pseudomolecules in the genome assembly of *Anania crocealis*, ilAnaCroc1.

INSDC accession	Chromosome	Length (Mb)	GC%
OX438880.1	1	29.34	36.0
OX438881.1	2	28.14	36.5
OX438882.1	3	27.85	36.0
OX438883.1	4	26.46	36.0
OX438884.1	5	25.28	36.0
OX438885.1	6	24.74	36.0
OX438856.1	7	24.57	36.0
OX438857.1	8	23.3	36.0
OX438858.1	9	23.18	36.0
OX438859.1	10	22.97	36.0
OX438860.1	11	22.87	36.5
OX438861.1	12	22.86	36.0
OX438862.1	13	22.57	36.0
OX438863.1	14	22.35	36.0
OX438864.1	15	21.14	36.0
OX438865.1	16	20.38	36.0
OX438866.1	17	20.21	36.0
OX438867.1	18	20.2	36.5
OX438868.1	19	20.04	36.5
OX438869.1	20	18.0	36.0
OX438870.1	21	17.62	36.5
OX438871.1	22	17.48	36.5
OX438872.1	23	14.85	36.0
OX438873.1	24	13.08	36.5
OX438874.1	25	12.99	36.5
OX438875.1	26	10.54	38.0
OX438876.1	27	10.1	38.0
OX438877.1	28	9.41	37.0
OX438879.1	Z	51.12	35.5
OX438878.1	MT	0.02	18.5

The estimated Quality Value (QV) of the final assembly is 67.7 with *k*-mer completeness of 100%, and the assembly has a BUSCO v5.3.2 completeness of 98.6% (single = 98.3%, duplicated = 0.3%), using the lepidoptera_odb10 reference set ( *n* = 5,286).

Metadata for specimens, barcode results, spectra estimates, sequencing runs, contaminants and pre-curation assembly statistics are given at https://links.tol.sanger.ac.uk/species/1100908.

## Genome annotation report

The *Anania crocealis* genome assembly (GCA_949315895.1) was annotated using the Ensembl rapid annotation pipeline ( Table 1; https://rapid.ensembl.org/Anania_crocealis_GCA_949315895.1/Info/Index). The resulting annotation includes 20,486 transcribed mRNAs from 20,293 protein-coding genes.

## Methods

### Sample acquisition and nucleic acid extraction

A male *Anania crocealis* (specimen ID NHMUK013698305, ToLID ilAnaCroc1) was collected by hand in Sandwich Bay Bird Observatory, England, UK (latitude 51.27, longitude 1.37) on 2021-09-24. The specimen was collected and identified by David Lees (Natural History Museum) and preserved by dry freezing at –80 °C.

The workflow for high molecular weight (HMW) DNA extraction at the Wellcome Sanger Institute (WSI) includes a sequence of core procedures: sample preparation; sample homogenisation; DNA extraction; HMW DNA fragmentation; and fragmented DNA clean-up. The sample was prepared for DNA extraction at the WSI Tree of Life laboratory: the ilAnaCroc1 sample was weighed and dissected on dry ice with tissue set aside for Hi-C sequencing ( Jay *et al.*, 2023). Tissue from the whole organism was disrupted using a Nippi Powermasher fitted with a BioMasher pestle ( Denton *et al.*, 2023a). HMW DNA was extracted in the WSI Scientific Operations core using the Automated MagAttract v2 protocol ( Oatley *et al.*, 2023). HMW DNA was sheared into an average fragment size of 12–20 kb in a Megaruptor 3 system with speed setting 31 ( Bates *et al.*, 2023). Sheared DNA was purified by solid-phase reversible immobilisation ( Strickland *et al.*, 2023): in brief, the method employs a 1.8X ratio of AMPure PB beads to sample to eliminate shorter fragments and concentrate the DNA. The concentration of the sheared and purified DNA was assessed using a Nanodrop spectrophotometer and Qubit Fluorometer and Qubit dsDNA High Sensitivity Assay kit. Fragment size distribution was evaluated by running the sample on the FemtoPulse system.

RNA was extracted from abdomen tissue of ilAnaCroc1 using the Automated MagMax™ *mir*Vana protocol ( do Amaral *et al.*, 2023). The RNA concentration was assessed using a Nanodrop spectrophotometer and Qubit Fluorometer using the Qubit RNA Broad-Range (BR) Assay kit. Analysis of the integrity of the RNA was done using the Agilent RNA 6000 Pico Kit and Eukaryotic Total RNA assay.

Protocols developed by the Tree of Life laboratory are publicly available on protocols.io ( Denton *et al.*, 2023b).

### Sequencing

Pacific Biosciences HiFi circular consensus DNA sequencing libraries were constructed according to the manufacturers’ instructions. Poly(A) RNA-Seq libraries were constructed using the NEB Ultra II RNA Library Prep kit. DNA and RNA sequencing was performed by the Scientific Operations core at the WSI on Pacific Biosciences SEQUEL II (HiFi) and Illumina NovaSeq 6000 (RNA-Seq) instruments. Hi-C data were also generated from remaining head and thorax tissue of ilAnaCroc1 using the Arima2 kit and sequenced on the Illumina NovaSeq 6000 instrument.

### Genome assembly, curation and evaluation

Assembly was carried out with Hifiasm ( Cheng *et al.*, 2021) and haplotypic duplication was identified and removed with purge_dups ( Guan *et al.*, 2020). The assembly was then scaffolded with Hi-C data ( Rao *et al.*, 2014) using YaHS ( Zhou *et al.*, 2023). The assembly was checked for contamination and corrected as described previously ( Howe *et al.*, 2021). Manual curation was performed using HiGlass ( Kerpedjiev *et al.*, 2018) and Pretext ( Harry, 2022). The mitochondrial genome was assembled using MitoHiFi ( Uliano-Silva *et al.*, 2023), which runs MitoFinder ( Allio *et al.*, 2020) or MITOS ( Bernt *et al.*, 2013) and uses these annotations to select the final mitochondrial contig and to ensure the general quality of the sequence.

A Hi-C map for the final assembly was produced using bwa-mem2 ( Vasimuddin *et al.*, 2019) in the Cooler file format ( Abdennur & Mirny, 2020). To assess the assembly metrics, the *k*-mer completeness and QV consensus quality values were calculated in Merqury ( Rhie *et al.*, 2020). This work was done using Nextflow ( Di Tommaso *et al.*, 2017) DSL2 pipelines “sanger-tol/readmapping” ( Surana *et al.*, 2023a) and “sanger-tol/genomenote” ( Surana *et al.*, 2023b). The genome was analysed within the BlobToolKit environment ( Challis *et al.*, 2020) and BUSCO scores ( Manni *et al.*, 2021; Simão *et al.*, 2015) were calculated.

Table 3 contains a list of relevant software tool versions and sources.

**Table 3.** Software tools: versions and sources.

Software tool	Version	Source
BlobToolKit	4.1.7	https://github.com/blobtoolkit/blobtoolkit
BUSCO	5.3.2	https://gitlab.com/ezlab/busco
Hifiasm	0.16.1-r375	https://github.com/chhylp123/hifiasm
HiGlass	1.11.6	https://github.com/higlass/higlass
Merqury	MerquryFK	https://github.com/thegenemyers/MERQURY.FK
MitoHiFi	2	https://github.com/marcelauliano/MitoHiFi
PretextView	0.2	https://github.com/wtsi-hpag/PretextView
purge_dups	1.2.3	https://github.com/dfguan/purge_dups
sanger-tol/genomenote	v1.0	https://github.com/sanger-tol/genomenote
sanger-tol/readmapping	1.1.0	https://github.com/sanger-tol/readmapping/tree/1.1.0
YaHS	1.2a	https://github.com/c-zhou/yahs

### Genome annotation

The BRAKER2 pipeline ( Brůna *et al.*, 2021) was used in the default protein mode to generate annotation for the *Anania crocealis* assembly (GCA_949315895.1) in Ensembl Rapid Release.

### Wellcome Sanger Institute – Legal and Governance

The materials that have contributed to this genome note have been supplied by a Darwin Tree of Life Partner. The submission of materials by a Darwin Tree of Life Partner is subject to the **‘Darwin Tree of Life Project Sampling Code of Practice’**, which can be found in full on the Darwin Tree of Life website here. By agreeing with and signing up to the Sampling Code of Practice, the Darwin Tree of Life Partner agrees they will meet the legal and ethical requirements and standards set out within this document in respect of all samples acquired for, and supplied to, the Darwin Tree of Life Project.

Further, the Wellcome Sanger Institute employs a process whereby due diligence is carried out proportionate to the nature of the materials themselves, and the circumstances under which they have been/are to be collected and provided for use. The purpose of this is to address and mitigate any potential legal and/or ethical implications of receipt and use of the materials as part of the research project, and to ensure that in doing so we align with best practice wherever possible. The overarching areas of consideration are:

    Ethical review of provenance and sourcing of the material

    Legality of collection, transfer and use (national and international)

Each transfer of samples is further undertaken according to a Research Collaboration Agreement or Material Transfer Agreement entered into by the Darwin Tree of Life Partner, Genome Research Limited (operating as the Wellcome Sanger Institute), and in some circumstances other Darwin Tree of Life collaborators.

## Data Availability

European Nucleotide Archive: *Anania crocealis*. Accession number PRJEB59304; https://identifiers.org/ena.embl/PRJEB59304 ( Wellcome Sanger Institute, 2023). The genome sequence is released openly for reuse. The *Anania crocealis* genome sequencing initiative is part of the Darwin Tree of Life (DToL) project. All raw sequence data and the assembly have been deposited in INSDC databases. Raw data and assembly accession identifiers are reported in Table 1.

## Author information

Members of the Natural History Museum Genome Acquisition Lab are listed here: https://doi.org/10.5281/zenodo.7139035.

Members of the Darwin Tree of Life Barcoding collective are listed here: https://doi.org/10.5281/zenodo.4893703.

Members of the Wellcome Sanger Institute Tree of Life Management, Samples and Laboratory team are listed here: https://doi.org/10.5281/zenodo.10066175.

Members of Wellcome Sanger Institute Scientific Operations: Sequencing Operations are listed here: https://doi.org/10.5281/zenodo.10043364.

Members of the Wellcome Sanger Institute Tree of Life Core Informatics team are listed here: https://doi.org/10.5281/zenodo.10066637.

Members of the Tree of Life Core Informatics collective are listed here: https://doi.org/10.5281/zenodo.5013541.

Members of the Darwin Tree of Life Consortium are listed here: https://doi.org/10.5281/zenodo.4783558.
