# Supplementary material for: The genome sequence of the Ochreous Pearl, Anania crocealis (Hübner, 1796)
Source: Wellcome Open Res. 2023 Dec 6;8:568. [Version 1] doi: 10.12688/wellcomeopenres.20573.1 (PMC11193085; doi:10.12688/wellcomeopenres.20573.1)
Supplement: Peer review history

# Reviewer report: Reviewer response for version 1

**DOI:** 10.21956/wellcomeopenres.22772.r86811

**Published:** 2024-06-21

**Authors:** Ruiqi Li

**Affiliations:** 1 University of Colorado Boulder, Boulder, Colorado, USA

Lees et al. present a genome assembly for the Ochreous Pearl, which appears to be of high quality and holds significant value for future research. Overall, the manuscript is well-prepared, but I have a few minor suggestions :

1. **Introduction**: The introduction provides a thorough overview of the natural history of the Ochreous Pearl and the divergence observed in its COI sequences. This sets a strong foundation for the study. However, the mention of the BOLD should be clarified. What is BOLD? Please include relevant citations.
2. The manuscript mentions adaptations for feeding on Asteraceae: Inuleae, but it lacks specifics. Could the authors elaborate on the types of adaptations observed? Additionally, why is feeding on Inuleae noteworthy? 
3. **Figure 4**: Figure 4 indicates that some scaffolds lack hits. Have the authors investigated the reasons for these missing hits? Could these scaffolds be contaminations or do they represent uncharacterized sequences? Further exploration or discussion of these findings would be valuable.

Are sufficient details of methods and materials provided to allow replication by others?

Yes

Is the rationale for creating the dataset(s) clearly described?

Yes

Are the datasets clearly presented in a useable and accessible format?

Yes

Are the protocols appropriate and is the work technically sound?

Yes

Reviewer Expertise:

Genomics

I confirm that I have read this submission and believe that I have an appropriate level of expertise to confirm that it is of an acceptable scientific standard.

# Reviewer report: Reviewer response for version 1

**DOI:** 10.21956/wellcomeopenres.22772.r75485

**Published:** 2024-03-12

**Authors:** Paula Escuer

**Affiliations:** 1 University of Neuchâtel, Neuchâtel, Switzerland

The authors presented the chromosome level genome sequence of a male individual of the Ochreous Pearl ( *Anania crocealis*). The final assembly shows a total length of 624.0 Mb condensed in 35 scaffolds with a scaffold N50 of 22.9 Mb. Mitochondrial assembly is also sequenced and available. The 99.95% of the assembly sequence is distributed in 29 chromosomal-level scaffolds, representing 28 autosomes plus the Z chromosome. The BUSCO pipeline indicated a high completeness of the genome with 98.6% of complete genes found, additional descriptive analyses are indicated in Table 1. Genome annotation identified 20,293 protein-coding genes. Standard bioinformatic pipelines were performed and software versions are indicated in Table 3. This high-quality assembly is a valuable tool to understand questions related to the evolution and adaptation of this genus and about the Lepidoptera order.

 While the methodological part and the data presented is robust, I would rephrase some parts of the introductory section, as sometimes can be a bit complicated to read:

 -   In the background, sometimes it is written UK, United Kingdom, England. It is better to standarise and use one of the terms or write United Kingdom (UK) and then use UK.

  

- I would rephrase the entire the first and second paragraph as following:

The Ochreous Pearl, *Anania crocealis* (Hübner, 1796), is a crambid moth, which boasts a wingspan measuring approximately 22–25 mm (Goater, 1986), with forewings spanning 11–12 mm (Sterling and Parsons, 2018). Its forewings display a pale yellow tone with minute reddish-brown orbicular and reniform stigmata, bounded by a convex basal transverse line and a distal transverse line that curves around the outer stigma. The hindwings are whitish-grey, with reddish-brown terminal lines in both wings. While resembling other Pyraustinae species found in the United Kingdom (UK), such as *Paratalanta hyalinalis* (Hübner, 1796) and the whiter Paracorsia repandalis ([Denis & Schiffermüller], 1775), the Ochreous Pearl differs in its unmarked hindwings. Like many pyraustines, it adopts a triangular resting posture.

 Adult moths fly in the UK between late June to August ( Goater, 1986; Parsons & Clancy, 2023), and It is considered as univoltine or occasionally bivoltine flying up to September or even November in the south of the UK ( Parsons & Clancy, 2023; Sterling & Parsons, 2018). The adult moth is mainly nocturnal but easily spotted around the foodplant during the day.

  

- I will also correct the next sentences:

*A. crocealis* is more or less common and widespread in southern parts of the United Kingdom, occurring more locally in Ireland, Wales, northwest England and Scotland, where **it is** scarce, as well as the Isle of Man and **the** Channel Islands ( Parsons & Clancy, 2023).

 - It was not specified if the RNA-seq reads were used for the annotation pipeline. It can clearly improve the quality of the annotation, so it is interesting to know if it was included.

Are sufficient details of methods and materials provided to allow replication by others?

Yes

Is the rationale for creating the dataset(s) clearly described?

Yes

Are the datasets clearly presented in a useable and accessible format?

Yes

Are the protocols appropriate and is the work technically sound?

Yes

Reviewer Expertise:

Genomics, Biodiversity, Speciation

I confirm that I have read this submission and believe that I have an appropriate level of expertise to confirm that it is of an acceptable scientific standard.
